# Bioaugmentation With a Consortium of Bacterial Sodium Lauryl Ether Sulfate-Degraders for Remediation of Contaminated Soils

**DOI:** 10.3389/fmicb.2021.740118

**Published:** 2021-09-22

**Authors:** Ludovica Rolando, Anna Barra Caracciolo, Paola Grenni, Livia Mariani, Jasmin Rauseo, Francesca Spataro, Gian Luigi Garbini, Andrea Visca, Luisa Patrolecco

**Affiliations:** ^1^Water Research Institute, National Research Council, Monterotondo, Italy; ^2^Institute of Polar Sciences, National Research Council, Monterotondo, Italy; ^3^Department of Ecological and Biological Sciences, Tuscia University, Viterbo, Italy

**Keywords:** anionic surfactant, foaming agents, spoil material, bioremediation, underground construction, bacterial consortium

## Abstract

The anionic surfactant sodium lauryl ether sulfate (SLES) is the main component of most commercial foaming agents (FAs) used in the excavation of highway and railway tunnels with Earth pressure balance-tunnel boring machines (EPB-TBMs). Several hundreds of millions of tons of spoil material, consisting of soil mixed with FAs, are produced worldwide, raising the issue of their handling and safe disposal. Reducing waste production and reusing by-products are the primary objectives of the “circular economy,” and in this context, the biodegradation of SLES becomes a key question in reclaiming excavated soils, especially at construction sites where SLES degradation on the spot is not possible because of lack of space for temporary spoil material storage. The aim of the present work was to apply a bacterial consortium (BC) of SLES degraders to spoil material excavated with an EPB-TBM and coming from a real construction site. For this purpose, the BC capability to accelerate SLES degradation was tested. Preliminary BC growth, degradation tests, and ecotoxicological evaluations were performed on a selected FA. Subsequently, a bioaugmentation experiment was conducted; and the microbial abundance, viability, and SLES concentrations in spoil material were evaluated over the experimental time (0.5, 3, 6, 24, 48, and 144 h). Moreover, the corresponding aqueous elutriates were extracted from all the soil samples and analyzed for SLES concentration and ecotoxicological evaluations with the bacterium *Aliivibrio fischeri*. The preliminary experiments showed the BC capability to grow under 14 different concentrations of the FA. The maximum BC growth rates and degradation efficiency (100%) were achieved with initial SLES concentrations of 125, 250, and 500 mg/L. The subsequent bioaugmentation of the spoil material with BC significantly (sixfold) improved the degradation time of SLES (DT_50_ 1 day) compared with natural attenuation (DT_50_ 6 days). In line with this result, neither SLES residues nor toxicity was recorded in the soil extracts showing the spoil material as a by-product promptly usable. The bioaugmentation with BC can be a very useful for cleaning spoil material produced in underground construction where its temporary storage (for SLES natural biodegradation) is not possible.

## Introduction

The anionic surfactant sodium lauryl ether sulfate (SLES) is the main component in most foaming agents (FAs) used in the excavation of highway and railway tunnels with Earth pressure balance-tunnel boring machines (EPB-TBMs) (Barra Caracciolo et al., 2017). FAs are used to change the mechanical properties and hydraulic behavior of soil, ensuring a malleable material that can be manageably excavated and transported to temporary areas (Peila, 2014; Peila et al., 2016). Due to the numerous tunneling projects currently in progress, several hundreds of millions of tons of soil debris (spoil material) are produced worldwide annually, raising the question of how to handle and dispose of them (Rahimzadeh et al., 2018; Rolando et al., 2020). Surfactants, including SLES, can be toxic for aquatic ecosystems if present at concentrations higher than the critical micellar one (Barra Caracciolo et al., 2017; Mariani et al., 2020; Rolando et al., 2020). The presence of SLES in spoil material can influence its recycling as a by-product; in fact, if the residual concentration of SLES is high, there can be toxic effects.

Previous experiments conducted on spoil material from tunnel excavation sites demonstrated that SLES was not toxic for terrestrial organisms (e.g., *Lepidium sativum* and *Eisenia fetida*) (Galli et al., 2019). However, in some cases, it showed detrimental effects on two aquatic species (*Pseudokirchneriella subcapitata* and *Aliivibrio fischeri*) (Grenni et al., 2018; Finizio et al., 2020; Patrolecco et al., 2020; Barra Caracciolo et al., 2021). Interestingly, the bacterium *A. fischeri* was shown to be very sensitive to SLES residues. In fact, a positive correlation between SLES concentrations and a toxic effect (inhibition of bioluminescence) on the bacterium was found in a 2-year monitoring of the spoil material from a highway construction site, making it possible to establish 2 mg/L as a “threshold value” of the toxic effect in the elutriates produced from spoil material (Mariani et al., 2020).

Reducing waste production and reusing by-products are the primary objectives of the “circular economy,” and in this context, the biodegradation of SLES becomes a key question in the safe reclaiming of excavated soils. Currently, tunnel debris can be used for refilling old quarries, road constructions, and green areas and as a raw material for industrial production (Bellopede and Marini, 2011).

Sodium lauryl ether sulfate has been shown to be a biodegradable compound in soils from tunnel excavation sites, but with highly variable degradation rates (Barra Caracciolo et al., 2019, 2021). In fact, concentrations of SLES in the range of 27–350 mg/kg showed variable half-lives (DT_50_) from 8 to 46 days (Finizio et al., 2020). These results were ascribable not only to the initial SLES concentrations but also to site-specific characteristics such as soil texture, depth, structure, mineralogy, microbial abundance, water content, and temperature, which influenced its biodegradation differently.

Although a natural attenuation (with no human intervention) of SLES is expected due to the environmental microorganisms present in the excavated soil, degradation times cannot always meet construction site requirements. In some cases, the spoil material can be temporarily stored at the construction site for the time needed for SLES biodegradation (Barra Caracciolo et al., 2019). This practice was successfully used in a recent tunnel construction site in Italy (Mariani et al., 2020). Unfortunately, in other cases, such as tunneling for a metro inside a city, an area for spoil material temporary storage is not available. In this case, the excavated materials have to be considered waste, requiring transportation, treatment, and disposal, with a significant increase in project costs and unnecessary landfill use (Barra Caracciolo et al., 2017; Rahimzadeh et al., 2018; Patrolecco et al., 2020).

In Italy, spoil material can be classified as a by-product if the chemical thresholds for some organic and inorganic contaminants (e.g., heavy metals, hydrocarbons C > 12) are not exceeded (Italian Decree, 2017). However, there are no SLES soil concentration limits (Annex 4 of the Italian Decree 120/2017) in EU and national legislation (Italian Decree, 2017). Consequently, the possibility that soil debris can be really considered a safe by-product is strongly related to SLES persistence and their residual concentrations, which in turn depend on abiotic and biotic site-specific conditions.

Sodium lauryl ether sulfate degradation is common in environmental bacteria able to resist the toxic effects of SLES and to degrade it (Barra Caracciolo et al., 2019). Several Gamma-*Proteobacteria* possess esterase enzymes that are able to break the SLES ester bond (Bornscheuer, 2002; Panda and Gowrishankar, 2005). In a recent previous work, a bacterial consortium (BC) capable of degrading completely pure SLES within 24 h was selected using enrichment cultures. The BC consisted of Gamma-*Proteobacteria* (99%), and the predominant (ca. 90%) genus was *Pseudomonas* (Rolando et al., 2020).

The aim of the present work was to test if adding the SLES pre-grown microbial culture (BC) to spoil material from a tunnel construction site enhanced the removal of the anionic surfactant residues, with potentially significant economic and environmental benefits.

## Materials and Methods

### Chemicals

Methanol and chloroform of high-performance liquid chromatography (HPLC) grade, hydrochloric acid (37%), sulfuric acid (98%), and methylene blue were purchased from VWR (Radnor, PA, United States). Sodium hydrogen carbonate and anhydrous sodium carbonate were obtained from CARLO ERBA Reagents (Milan, Italy). SLES of technical grade purity was from BOC Sciences (Shirley, NY, United States). The stock solution of SLES (1,000 mg/L) was prepared in methanol and stored at −20°C. The dilution of this stock solution was performed using ultrapure water. Ultrapure water (18 MΩ⋅ cm quality) was obtained using a Milli-Q system Millipore (Bedford, MA, United States). Diatomaceous earth was purchased from Thermo Fisher Scientific Inc. (Waltham, MA, United States).

### Analytical Determination of Sodium Lauryl Ether Sulfate in the Foaming Agent

The SLES concentration in FA solutions, used for the preliminary BC growth and degradation test described in the *Chemicals* section, was determined by applying the methylene blue active substances (MBAS) method^1^. This method includes three consecutive chloroform extractions of the ionic-pair reaction between SLES and methylene blue. Subsequently, the absorbance of the SLES–MBAS complex was measured with spectrophotometry at a wavelength of 650 nm (Lambda 25UV–VIS spectrophotometer, PerkinElmer, Waltham, MA, United States). Finally, the SLES concentration was calculated using the equations obtained with the standard calibration curve (in the range from 0.05 to 4 mg/L of SLES), as previously described (Barra Caracciolo et al., 2019; Patrolecco et al., 2020). The limit of detection (LOD), calculated following the IUPAC method (IUPAC, 1999), was 0.013 mg/L.

### Preparation of Elutriates From Soil Samples

The elutriates (soil water extracts) were produced from soil samples in a 1:10 (solid/liquid) ratio, as reported in Grenni et al. (2018), following the procedure described in UNI EN 12457-4:2004 (UNI EN, 2004). This standard procedure was used to simulate possible leaching of SLES from soil to water. Briefly, an aliquot (10 g) of fresh soil sample was put into a 250-ml bottle, and the calculated amount of distilled water (taking into account the moisture of the soil sample) was added. The suspension was shaken for 24 h at 20°C in the dark and settled, and the supernatant was then centrifuged for 15 min at 9,000 rpm.

### Analytical Determination of Sodium Lauryl Ether Sulfate in Soil and Elutriates

Sodium lauryl ether sulfate was extracted from soil samples with the pressurized liquid extraction (PLE) technique (Buchi mod. E-916; Cornaredo, Milan, Italy), using methanol as the extraction solvent and following the operative conditions reported in Pescatore et al. (2020). The PLE extracts and aqueous elutriates were analyzed for SLES content using the spectrophotometric MBAS method (see section “Analytical Determination of Sodium Lauryl Ether Sulfate in the Foaming Agent”). The PLE recovery was 96.5 ± 1.6%.

### Preliminary Bacterial Consortium Growth and Degradation Test on the Foaming Agent Selected

The previously isolated BC, capable of degrading pure SLES in 24 h and using it as the only carbon source (Rolando et al., 2020), was initially tested on a commercial FA consisting of a water solution of SLES (16%). FA was selected because it is one of the most common commercial products used in Italy and Europe for tunnel excavation with EPB-TBMs.

The capability of the BC to grow on different FA amounts was tested. For this purpose, various FA water solutions were prepared to obtain 14 SLES concentrations (0.5 mg/L, 1 mg/L, 2 mg/L, 4 mg/L, 8 mg/L, 16 mg/L, 31 mg/L, 62.5 mg/L, 125 mg/L, 250 mg/L, 500 mg/L, 1 g/L, 2 g/L, and 4 g/L). For each concentration, three replicates were considered. A control with only a mineral medium and a control with a mineral medium (MB1) and the BC were also set up. The bacterial growth was measured (every 15 min for 24 h) in terms of optical density (OD, 600_nm_), using a Multiskan Sky Microplate Spectrophotometer (Thermo Fisher Scientific). A 96-well plate was used to incubate the cultures at 37°C with a background shaking (180 rpm) and then read with the spectrophotometer for BC growth.

The capability of the BC to degrade the FA was then verified with the five concentrations that showed the highest growth rates (125 mg/L, 250 mg/L, 500 mg/L, 1 g/L, 2 g/L, and 4 g/L). Culture flasks (100 ml, three replicates for each condition) containing MB1 and FA solutions were incubated at 28°C in the dark and maintained on a rotary shaker at 130 rpm. A control with only MB1 and a control with MB1 and the BC were also set up. Cell growth (OD) and SLES concentration (% residual SLES concentration) were measured at 0, 6, 24, and 48 h.

### Ecotoxicological Evaluation (EC_20_ and EC_50_) of the Foaming Agent

The intrinsic toxicity of the FA was evaluated using the bioluminescent bacterium *A. fischeri*, following the UNI EN ISO 11348-3:2019 protocol, reported in detail in section “*Aliivibrio fischeri* Acute Toxicity Test.” The test with *A. fischeri* was used because previous studies have demonstrated it to be very sensitive to SLES residues in spoil material (Grenni et al., 2018; Mariani et al., 2020; Patrolecco et al., 2020).

The FA toxicity was expressed as the effective concentration (EC), i.e., the concentration that causes an effect (bioluminescence inhibition in percentage) on 20% (EC_20_) or 50% (EC_50_) of the organisms tested.

A higher EC value corresponds to a lower ecotoxicological effect. The EC_20_ and EC_50_ were determined using the Basic Test (81.9%) performed three times. The bacterium *A. fischeri* was exposed to various FA concentrations prepared with subsequent dilutions (using distilled water) from an FA stock solution (105 mg/L). Seven diluted solutions (in the range 1.34–86.00 mg/L) of the FA were used. The EC_20_ and EC_50_ were statistically estimated (Microtox Omni^®^ software V 4.2, Milan, Italy). Based on the results obtained (see sections “Preliminary Bacterial Consortium Growth and Degradation Test on the Foaming Agent Selected” and “Ecotoxicological Evaluation (EC_20_ and EC_50_) of the Foaming Agent”), the commercial product FA was used for the subsequent bioaugmentation and ecotoxicological experiments, as described in the following paragraphs.

### Bioaugmentation Experiment With Spoil Material From the Construction Site

The soil samples consisted of spoil material directly obtained from the EPB-TBM operating in a railway tunnel construction in Southern Italy. The experimental set consisted of glass beakers (2,000-ml capacity) filled with about 800 g of the excavated soil. The spoil material consisted of a silty clay soil conditioned with the commercial FA containing SLES (16%). The FA treatment ratio was 1.017 L/m^3^, which corresponded to an expected SLES concentration of 130 mg/kg. The soil water content was 60%, corresponding to 70% of the maximum soil water-holding capacity.

The BC was seeded on the spoil material to test its capability to accelerate the FA degradation.

The overall experimental conditions were as follows (three replicates for each condition):

- Soil batches with the FA and the BC: **FA + BC soil**.
- Soil batches with only the FA: **FA soil**.
- Soil batches with only the BC: **BC soil**.
- Untreated soil batches: **Control**.

The overall experimental set was maintained at room temperature 20 ± 2°C and kept open in order to simulate the temporary storage of the spoil material at the construction site. At selected times (0.5, 3, 6, 24, 48, and 144 h), three subsamples of soil from each batch were collected to analyze the microbial community [4’,6-diamidino-2-phenylindole (DAPI) counts and Live/Dead] and the concentration of SLES over time. Moreover, from each soil sample, the corresponding aqueous elutriate was produced (see section “Preparation of Elutriates From Soil Samples”) and analyzed for SLES concentration and ecotoxicological evaluations with the bacterium *A. fischeri*. All results refer to dry weight.

### Preparation of the Bacterial Inoculum

The BC was cultured in a mineral medium [MB1: 0.8 g/L of K_2_HPO_4_, 0.2 g/L of KH_2_PO_4_, 0.05 g/L of CaSO_4_.2H_2_O, 0.5 g/L of MgSO_4_.7H_2_O, 0.01 g/L of FeSO_4_, and 1 g/L of (NH_4_)_2_SO_4_] with 250 mg/L of SLES and maintained at 28°C overnight. From the overnight static culture, the bacterial cells were centrifuged (9,000 rpm for 2 min) and re-suspended in distilled water and the latter plus the FA. Before the spoil material was seeded, the bacterial abundance and viability of the BC were also measured using the DAPI count and Live/Dead methods, respectively. The bacterial abundance was 2.19 × 10^7^ ± 1.22 × 10^6^ cell/ml with a 90.7 ± 0.2% of live cells.

### Total Bacterial Abundance and Cell Viability by Epifluorescence Direct Methods

A fluorescence microscope (Leica DM 4000B, Leica Microsystems GmbH, Wetzlar, Germany) was used to observe and count the total bacterial abundance and cell viability. Briefly, total bacterial abundance (No. cells/g soil) was obtained (three subsample replicates) using aliquots of samples (ranging from 0.25 to 1 ml), fixed with formaldehyde (2% final concentration), and filtered through a 25-mm black polycarbonate membrane with a porosity of 0.2 μm (Whatman) using a gentle vacuum (<0.2 bar). The filters were treated with DAPI as described in detail elsewhere (Barra Caracciolo et al., 2005a,b). The total number of bacterial cells using DAPI can detect all microbial cells in a sample regardless of their physiological status and metabolic activity.

Cell viability (% live cells/live + dead) was assessed (three subsample replicates) using aliquots of fresh samples (the same volumes used for total bacterial abundance), which were filtered through the same filters as for the DAPI counts. The filters were treated with two fluorescent dyes, SYBR Green II and propidium iodide, as described in detail elsewhere (Grenni et al., 2009). This method can detect the viability of microorganisms, because propidium iodide dye can only enter dead or damaged cells, and they appear red under a fluorescence microscope. Finally, the abundance of viable cells (No. viable cells/g soil) was calculated by multiplying the total bacterial abundance (obtained by DAPI counting) by cell viability.

### *Aliivibrio fischeri* Acute Toxicity Test

The acute toxicity test with *A. fischeri* was performed using a Microtox^®^ analyzer (Model 500; Ecotox LDS, Milan, Italy) in accordance with the UNI EN ISO 11348-3: 2019 standard protocol (UNI EN, 2019). This test is based on the inhibition of the luminescence naturally emitted by the marine bacterium *A. fischeri* after its exposure to a toxic substance. Light output of the test organism, compared with a blank (toxic-free solution: distilled water containing 22% NaCl), was measured at least three times after each exposure period (5, 15, and 30 min). The difference in light output (between the sample and the blank) was ascribed to the matrix (elutriate) effect on the bacterium. The effect was calculated as a percentage of inhibition, using specific software (Microtox Omni^®^ software V 4.2, Milan, Italy). Before the tests were carried out, the pH value of each elutriate was recorded and eventually corrected (range 6.0–8.0) using an HCl 0.1 M solution (Scheerer et al., 2006), as required by the standard procedures. The coefficient of variation (CV%: standard deviation/mean × 100) as a validity criterion (has to be <20%) was also calculated. The bacterial response (% bioluminescent inhibition) was considered toxic if it was more than 20% (Persoone et al., 2003), in accordance with the UNI EN ISO 11348-3:2019 protocol (UNI EN, 2019).

### Statistical Analysis

Any differences among data were evaluated using the Kruskal–Wallis test. The relationship between variables was calculated using a linear regression model. All statistical analyses were performed using R software version 4.1.0. Average and standard errors were estimated from three technical replicates of three biological replicates using MS Excel version 16.16.27. All figures were made using MS Excel version 16.16.27.

## Results

### Preliminary Bacterial Consortium Growth and Degradation Test on the Foaming Agent Selected

**FIGURE 1:**
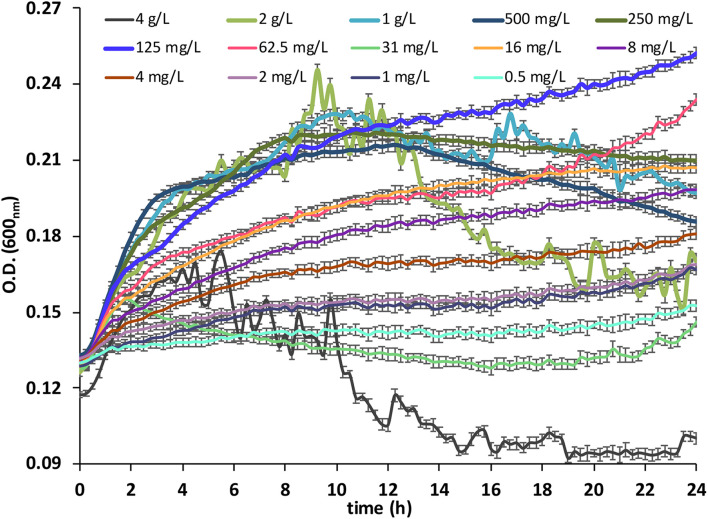
Bacterial consortium growth measured as optical density (OD) at 600_nm_, under 14 concentrations (from 0.5 mg/L to 4 g/L) of SLES. The vertical bars represent the standard errors. SLES, sodium lauryl ether sulfate.

A significant growth in the BC was observed with all 14 concentrations tested (Figure 1), confirming that the anionic surfactant SLES contained in the FA was a suitable carbon source.

The maximum BC growth rates are reported in Table 1. The growth rates were calculated with the following formula (Monod, 1949):


(1) $$\mu =\frac{log_{10}OD_{x}-log_{10}OD_{0}}{t_{x}-t_{0}}$$

where OD_*x*_, optical density measured at sampling time; OD_0_, optical density measured at time 0; t_*x*_, sampling time; and t_0_, time 0.

**TABLE 1:** Maximum growth rates of the bacterial consortium grown under 14 different SLES concentrations.

SLES concentration (mg/L)	4,000	2,000	1,000	500	250	125	62.5	31	16	8	4	2	1	0.5
μ_max_ (h^–1^)	0.037	0.047	0.043	0.044	0.043	0.043	0.037	0.035	0.037	0.023	0.018	0.014	0.013	0.010

*SLES, sodium lauryl ether sulfate.*

*μ_max_ = maximum growth rate during the exponential phase.*

The five SLES concentrations (125 mg/L, 250 mg/L, 500 mg/L, 1 g/L, and 2 g/L), which showed significantly (*p* < 0.01, Kruskal–Wallis test) higher BC growth rates, were used for the subsequent degradation experiment.

**FIGURE 2:**
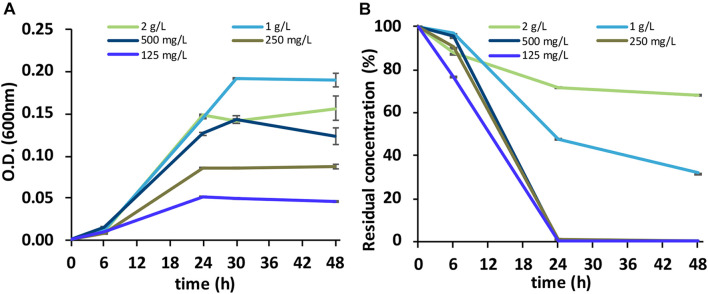
**(A)** Growth of the BC (OD at 600_*nm*_) on the foaming agent containing SLES. **(B)** Biodegradation of SLES (residual concentration) at five concentrations (125 mg/L, 250 mg/L, 500 mg/L, 1 g/L, and 2 g/L). The vertical bars represent the standard errors. BC, bacterial consortium; SLES, sodium lauryl ether sulfate.

The BC was capable of growing with all five SLES concentrations tested (Figure 2A). However, a complete degradation of the anionic surfactant was observed at 48 h only with the 125, 250, and 500 mg/L concentrations (Figure 2B).

### Ecotoxicological Evaluation (EC_20_ and EC_50_) of the Foaming Agent

The dose–response relationship between the FA concentration and the bioluminescence inhibition (%) after 30 min of exposure of the bacterium *A. fischeri* was determined. The EC_20_ and EC_50_ average values for the FA were 3.66 ± 0.72 (corresponding to 0.59 mg/L of SLES) and 10.34 ± 1.20 (corresponding to 1.66 mg/L of SLES) mg/L, respectively. In both cases, the CV% was less than 20%.

### Bioaugmentation Experiment

#### Total Microbial Abundance and Cell Viability in Soil

**FIGURE 3:**
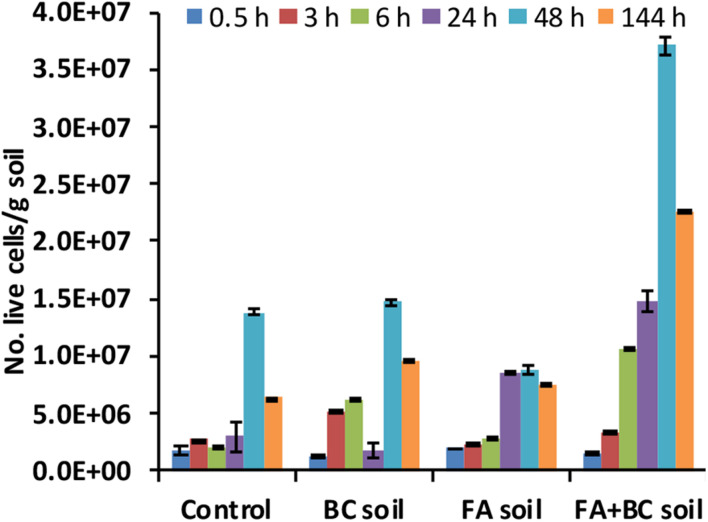
Number of live cells (No. live cells/g soil) evaluated using direct fluorescence methods at different experimental times (0.5–144 h) in the various conditions. Control: untreated soil batches; BC soil: soil batches with only the bacterial consortium; FA soil: soil batches with only the foaming agent; FA + BC soil: soil batches with the foaming agent and the bacterial consortium. The vertical bars represent the standard errors.

The initial microbial abundance (expressed as No. of live cells/g soil) in the spoil material used for the bioaugmentation experiment was similar in all conditions (1.65 × 10^6^ ± 1.24 × 10^5^). FA treatment did not have any initial detrimental effect on this microbiological parameter. An increasing trend in microbial numbers was observed between 0.5 and 48 h. A significantly (*p* < 0.01, Kruskal–Wallis test) higher abundance was observed in the bioaugmented soil (FA + BC soil), with a peak at 48 h (Figure 3).

#### Sodium Lauryl Ether Sulfate Concentration in Soil and Elutriates

**FIGURE 4:**
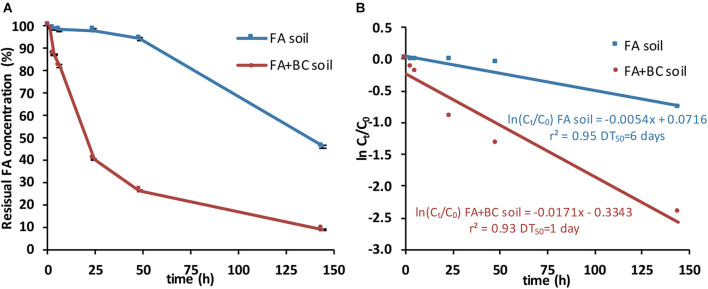
SLES degradation in soil batches with only the foaming agent (FA soil) and soil batches with the foaming agent and the bacterial consortium (FA + BC soil). **(A)** Residual concentrations of SLES expressed in percentage (%) over the experimental time (144 h). **(B)** Plot of ln(C_t_/C_0_) versus time for calculating the theoretical SLES DT_50_ (days) by the linear regression equations. The vertical bars represent the standard errors. SLES, sodium lauryl ether sulfate.

The residual concentrations of SLES measured over the experimental time (0.5, 3, 6, 24, 48, and 144 h) in the spoil material and in the corresponding elutriates produced are shown in Figure 4 and Table 2, respectively. The initial SLES amounts in the spoil material (FA soil) and in the bioaugmented soil (FA + BC soil) were 136 ± 3.6 and 132.3 ± 2.8 mg/kg, respectively.

**TABLE 2:** SLES concentrations (mg/L) in elutriates obtained from the soil samples of the conditioned batches (FA soil and FA C BC soil) over the experimental time; SE = standard error.

	SLES (mg/L) in elutriates			
	0.5 h	24 h	48 h	144 h
FA soil	6.0 ± 0.01	5.4 ± 0.01	2.2 ± 0.00	1.2 ± 0.00
FA + BC soil	5.2 ± 0.00	1.0 ± 0.01	0.2 ± 0.00	<LOD^a^

*SLES, sodium lauryl ether sulfate;*

*FA, foaming agent;*

*BC, bacterial consortium.*

*^*a*^LOD, limit of detection.*

In the bioaugmented soil (FA + BC soil), 91% of SLES was degraded at 144 h. At the same sampling time, in the absence of the BC, only 54% of SLES was degraded (FA soil; Figure 4A).

The degradation pathways followed first-order kinetics, and the theoretical values of the disappearance time of 50% of the initial SLES concentrations (DT_50_) were calculated from correlations (*r*^2^ = 0.93 for FA + BC soil and 0.95 for FA soil; *p*-value < 0.01) between concentrations [expressed as ln(C_t_/C_0_), where C_t_, SLES concentration at the sampling time and C_0_, SLES concentration at day 0] versus time (Figure 4B).

The SLES concentration in the elutriates reflected that of the soil samples (Table 2). To the SLES initial concentrations in the spoil material of 136 and 132.3 mg/kg corresponded 6.0 and 5.2 mg/L of SLES in the elutriates produced from FA soil and FA + BC soil conditions, respectively.

In line with the SLES degradation observed in soil, the anionic surfactant also decreased over time in the corresponding water extract. In the bioaugmented soil, significantly lower SLES residues were detected; for example, at 24 h, a SLES concentration of 1 mg/L was found in FA + BC soil and at the same time of 5.4 mg/L in FA soil. At the end of the bioaugmentation experiment, no SLES residues were found in the FA + BC soil condition.

#### *Aliivibrio fischeri* Acute Toxicity Test

**FIGURE 5:**
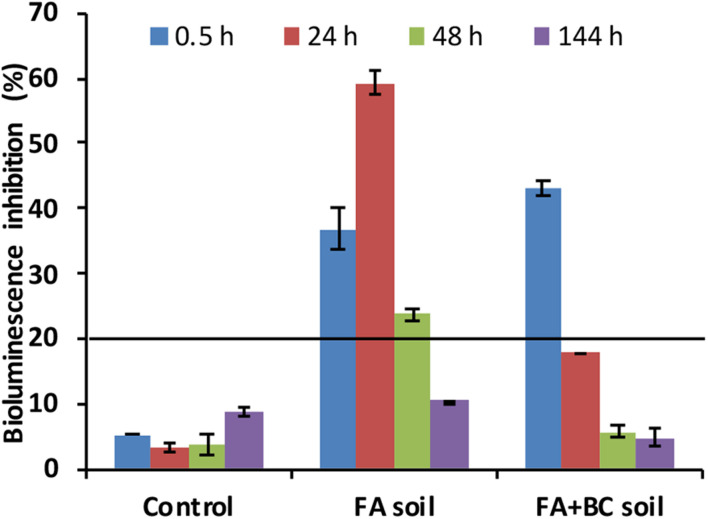
Bioluminescence inhibition (%) of *Aliivibrio fischeri* bacterium exposed for 30 min to elutriates from FA, FA + BC soil, and Control at selected experimental times (0.5, 24, 48, and 144 h) of the bioaugmentation experiment. Control: untreated soil batches; FA soil: soil batches with only the foaming agent; FA + BC soil: soil batches with the foaming agent and the bacterial consortium. The bars represent the standard errors. The black line represents the threshold of toxicity (20%).

The *A. fischeri* test (Figure 5) was performed on water extracts (elutriates) of unconditioned (Control) and conditioned (FA soil and FA + BC soil) soils from the bioaugmentation experiment. The test was executed on samples collected at selected times (0.5, 24, 48, and 144 h).

In the Control condition (as expected), the bacterial bioluminescence was not inhibited. At the start (0.5 h) of the experiment, a toxic effect (bioluminescence inhibition >20%) was observed in elutriates of both FA and FA + BC soil; at the subsequent sampling, FA + BC soil elutriates did not show any toxicity for the *A. fischeri* bacterium. On the other hand, a toxic effect (bioluminescence inhibition >20%) was observed in FA soil until 48 h.

All validity criteria for the test were met, and all the data reported can therefore be considered valid.

## Discussion

The aim of the present work was to test the capability of a previously isolated BC to enhance SLES degradation, in FA-conditioned soil collected from a tunnel construction site, in order to verify its possible use for routine bioaugmentation purposes. Bioaugmentation is a green technology used successfully for bioremediation of soil and water from several contaminants (e.g., petroleum hydrocarbons and pesticides) (Gentry et al., 2004; Abatenh et al., 2017; Arora, 2018). It consists in adding exogenous microbial populations or autochthonous ones (as in this work) with the catabolic potential to remove specific pollutants, such as pesticides (Plangklang and Reungsang, 2011; Sun et al., 2020). One advantage of bioaugmentation is that the degradation process starts as soon as specific microbial degraders are introduced (Wu et al., 2019). The success of bioaugmentation depends on the ability of the inoculated microorganisms to continue their activity in the environment as long as necessary for contaminant removal. However, if the degradation times are relatively long (such as in the case of pesticides), competitive interactions with non-degrading microbial populations can occur, and specific environmental conditions can limit bioaugmentation efficiency during *in situ* remediation (Hibbing et al., 2010; Asok and Jisha, 2012; Stelting et al., 2014; Cavinato et al., 2017).

The fact that in our work the bacterial populations were isolated from the same soil and that SLES removal times were very low made this bioremediation strategy an example of a real nature-based solution.

Although there are several works (Cycoń et al., 2017; Nur Zaida and Piakong, 2018; Tondera et al., 2021) dealing with contaminant removal by bioaugmentation, most of them have been performed using culture media and/or in liquid media and at concentrations which do not reflect those found in real contamination scenarios (Silva et al., 2004). There are few works in which adding microbial strains to soil improved degradation of organic contaminants in field studies or directly in environmental soil samples. Bioaugmentation can be applied using single microbial populations or a microbial consortium. For example, microbial consortia have been reported to degrade oils in polluted soil (Silva-Castro et al., 2012), in waste lubricating oil (Bhattacharya et al., 2015), in seawater (Dellagnezze et al., 2016), and in petroleum hydrocarbon in contaminated groundwater (Poi et al., 2018) and triazines in water and soil (Nasseri et al., 2014; Sagarkar et al., 2014). Triazine degradation has been performed in several bioaugmentation experiments with concentrations of these herbicides much higher than those commonly found in soil, such as in the study by Silva et al. (2004). However, in a recent work, Chen et al. (2021) showed the capability of a single bacterial population to degrade atrazine in a real contaminated soil (Chen et al., 2021).

Most works on anionic surfactants are aimed at evaluating biodegradation of sodium dodecyl sulfate, linear alkylbenzene sulfonate, and SLES in activated sludge (Hosseini et al., 2007), industrial wastewater (Karray et al., 2016; Fedeila et al., 2018), municipal wastewater (de Faria et al., 2019), and wastewater treatment plants (Paulo et al., 2017), because they are contaminants widely used in cosmetics, cleaning products, and personal care products (Barra Caracciolo et al., 2017; Hashim et al., 2018).

On the other hand, there have been no bioaugmentation experiments for remediating SLES, except for the work by Dhouib et al. (2003). These authors found a bacterial population of Gamma-*Proteobacteria* (*Citrobacter braakii*) that was able to degrade high concentrations of SLES in an enrichment culture using wastewater samples from a cosmetic plant as the inoculum. This work supports our results and the role of Gamma-*Proteobacteria* class in removing this anionic surfactant. Interestingly, in our study, where the soil came from a real construction site and the surfactant concentrations (130 mg/kg) were those used for the excavation, the bioaugmentation of the spoil material with the BC significantly (sixfold) improved the natural degradation time of SLES. Moreover, from an applied perspective, using a microbial consortium rather than a pure culture is more advantageous because it provides the metabolic diversity and robustness needed for field applications (Tyagi et al., 2011).

Finally, the ecotoxicological results confirmed the depollution of the soil (SLES in elutriates did not exert any toxicological effect) and the high sensitivity of *A. fischeri* to SLES residues higher than 2 mg/L (before SLES degradation), in line with the findings of previous works (Mariani et al., 2020; Patrolecco et al., 2020). The use of this ecotoxicological test in supporting chemical analysis is very powerful because it is an effective tool that can evaluate an overall matrix toxicity including chemicals (e.g., possible metabolites and/or unknown elements present in soil) non-directly analyzed. Other studies used *A. fischeri* as an effective screening test for soil samples (Parvez et al., 2006; Abbas et al., 2018).

## Conclusion

Bioaugmentation with the BC identified at a construction site makes it possible to remove anionic surfactant residues and clean up spoil material in a few hours, ensuring a safe by-product and saving execution time and overall costs for the tunneling industry. To our knowledge, this study is the first using a SLES-degrading BC for bioaugmentation purposes in a contaminated soil from a real environmental scenario. This remediation strategy is a promising low-cost and nature-based solution, for a prompt and safe reuse of the spoil material, avoiding its temporary storage and unnecessary waste production and transfer to landfills. This technique could be used, e.g., in the case of metro tunneling inside a city or through mountains along a sea coast, where the lack of space prevents temporary storage of the spoil material for its natural attenuation. The overall results show how a diversified approach involving chemical, microbiological, and ecotoxicological assessments of polluted soil can improve our understanding of the biodegradability of pollutants in bioremediation strategies. Further studies are in progress for finding the best practice (e.g., alginate microspheres) for seeding the BC on a large scale in construction sites.

## Data Availability Statement

The original contributions presented in the study are included in the article/supplementary material, further inquiries can be directed to the corresponding author/s.

## Author Contributions

LR, AB, PG, LM, JR, FS, GG, AV, and LP: methodology, formal analysis, and validation. LR and AB: writing, original draft preparation, and editing. AB, PG, and LP: supervision. AB: funding acquisition. All authors have read and agreed to the published version of the manuscript.

## Conflict of Interest

The authors declare that the research was conducted in the absence of any commercial or financial relationships that could be construed as a potential conflict of interest.

## Publisher’s Note

All claims expressed in this article are solely those of the authors and do not necessarily represent those of their affiliated organizations, or those of the publisher, the editors and the reviewers. Any product that may be evaluated in this article, or claim that may be made by its manufacturer, is not guaranteed or endorsed by the publisher.

## Footnotes

1
https://www.nemi.gov/methods/method_summary/7612/
